# Simultaneous Upregulation of Elastolytic and Elastogenic Factors Are Necessary for Regulated Collateral Diameter Expansion

**DOI:** 10.3389/fcvm.2021.762094

**Published:** 2022-01-12

**Authors:** Elizabeth Andraska, Nolan Skirtich, Dylan McCreary, Rohan Kulkarni, Edith Tzeng, Ryan McEnaney

**Affiliations:** ^1^Division of Vascular Surgery, Department of Surgery, University of Pittsburgh Medical Center, Pittsburgh, PA, United States; ^2^University of Pittsburgh, School of Medicine, Pittsburgh, PA, United States

**Keywords:** arteriogenesis, elastic fiber, extracellular matrix, remodeling, collateral, arterial occlusive disease

## Abstract

**Background:** During arteriogenesis, outward remodeling of the arterial wall expands luminal diameter to produce increased conductance in developing collaterals. We have previously shown that diameter expansion without loss of internal elastic lamina (IEL) integrity requires both degradation of elastic fibers and LOX-mediated repair. The aim of this study was to investigate the expression of genes involved in remodeling of the extracellular matrix (ECM) using a model of arteriogenesis.

**Methods:** Sprague-Dawley rats underwent femoral artery ligation with distal arteriovenous fistula (FAL + AVF) placement. Profunda femoral arteries (PFA) were harvested for analysis at various time points. Serum desmosine, an amino acid found exclusively in elastin, was evaluated with enzyme-linked immunosorbent assay (ELISA) as a marker of tissue elastolysis. Tissue mRNA isolated from FAL + AVF exposed PFAs was compared to the contralateral sham-operated using qPCR. HCAECs were cultured under low shear stress (8 *dyn*·*s/cm*^2^) for 24 h and then exposed to high shear stress (40 *dyn*·*s/cm*^2^) for 2–6 h. Primers used included FBN-1, FBN-2, Timp-2, LOX-1, Trop-E, Cath-K, Cath-S, MMP-2, MMP-9, FBLN-4, and FBLN-5 and were normalized to GAPDH. mRNA fold changes were quantified using the 2-ΔΔCq method. Comparisons between time points were made with non-parametric ANOVA analysis with Bonferroni adjustment.

**Results:** PFAs showed IEL reorganization during arteriogenesis. Serum desmosine levels are significantly elevated at 2 days and one week, with a return to baseline thereafter (*p* < 0.01). Expression of ECM structural proteins (FBN-1, FBN-2, FBLN-4, FBLN-5, Tropoelastin, TIMP-2, LOX-1) and elastolytic proteins (MMP-2, MMP-9, Cathepsin S, Cathepsin K) exhibited an early peak (*p* < 0.05) relative to sham PFAs. After two weeks, expression returned to baseline. HCAECs demonstrated upregulation of FBN-2, FBLN-5, LOX-1 and Trop-E at 4 h of high shear stress, as well as elastolytic protein MMP-2.

**Conclusions:** Elastin degradation begins early in arteriogenesis and is mediated by local upregulation of elastolytic genes. Elastolysis appears to be simultaneously balanced by production of elastic fiber components which may facilitate stabilization of the IEL. Endothelial cells are central to initiation of arteriogenesis and begin ECM remodeling in response to altered shear stress.

## Introduction

Arterial occlusive disease (AOD) affects >200 million people worldwide and can be present in all arterial beds. AOD remains the leading cause of death and disability in Western nations (1). Current therapies depend upon invasive revascularization techniques ranging from endovascular angioplasty to open surgical bypass. Unfortunately, many individuals suffering from advanced AOD are poor candidates for invasive revascularization and currently no effective medical therapy exists (2).

Collateral arteries develop through a process known as arteriogenesis, largely among pre-formed arterial interconnections that are remote from the effects of ischemia. Arteriogenesis occurs in response to changes in fluid shear stresses that follow conductance arterial occlusion. Functional collateral arteries maintain perfusion of tissues (3–5). Despite their importance, spontaneously formed collateral artery networks are not sufficient to replace an occluded conductance artery. Evidence suggests, however, that collateral arterial conductance can potentially be driven farther, which may indicate an opportunity for therapeutic development (6, 7). Maximizing collateral network capacity by enhancing arteriogenesis would hold promise for treatment of AOD.

Early after conductance arterial occlusion, increased fluid shear stress becomes recognized at the endothelial cell (EC) level within vessels bridging arterial territories, leading to EC activation. This is followed by recruitment of various inflammatory cells and subsequent cytokine production (8, 9). Proteases and elastases, including matrix metalloproteinases (MMPs), are released within the vessel wall which permits outward diameter enlargement by releasing the elastic and collagen fiber constraints (10, 11). Elastin degradation is known to occur during arteriogenesis and histologically, the internal elastic lamina (IEL) has been described as becoming fragmented and transiently disappearing in developing collateral arteries (12). Maintenance of elastic fiber integrity is an important part of outward arterial remodeling and likely preserves functional structure of the extracellular matrix (ECM) (13). However, the balance of proteolysis with structural repair of this restructuring has not been characterized and is the focus of our study.

We used an animal model of enhanced arteriogenesis which drives exaggerated outward remodeling of arterial tissues. We hypothesis that arterial expansion requires (1) partial disruption of elastic tissue and (2) nearly simultaneous repair of elastic tissue to stabilize the vessel wall and prevent loss of elastic fiber integrity. We hypothesize that the endothelium initiates this process as it is the only tissue poised to respond to hemodynamic changes.

## Methods

### Animal Models

All animal surgeries were approved by the Institutional Animal Care and Use Committee at the University of Pittsburgh (Protocol # 19095696) and were performed in accordance with the NIH Guide for the Care and Use of Laboratory Animals. 32 male Sprague Dawley rats (Envigo RMS; Frederick, MD) underwent a modified model of hind limb ischemia that has been previously described (7). Briefly, an arteriovenous fistula (AVF) is created between the common femoral artery (CFA) and the common femoral vein (CFV). The CFA is then ligated (femoral artery ligation, FAL) proximally to the arteriovenous anastomosis. For sham operations, the CFA was exposed but no FAL or AVF was performed. Sham operations were performed at the time of the FAL+AVF. Animals were sacrificed either immediately, or after 2 days, 1, 2, 4, 8, or 12 weeks. FAL + AVF procedures were performed on the left limb, whereas sham operations were performed on the right limb.

The intent of this study is to compare an actively remodeling artery to its contralateral counterpart that is not undergoing remodeling. The profunda artery isolated from a sham-operated hindlimb was chosen to form the internal control for each individual rat undergoing a contralateral FAL + AVF. The FAL + AVF model was utilized because we can consistently observe substantial outward remodeling (>100% diameter increase) of a surgically accessible and anatomically consistent vessel in the profunda. This FAL + AVF model has been previously compared to FAL alone with regards to the degree of morphologic remodeling observed (7). We have reported that FAL alone does not result in consistent outward diameter increases in the profunda when compared to sham operation. However, the addition of the AVF does result in consistent and reliable diameter increases with outward remodeling (7). Therefore, FAL + AVF vs. sham operated limb was chosen as the most appropriate model compared to FAL + AVF vs. FAL alone.

**Figure 1:**
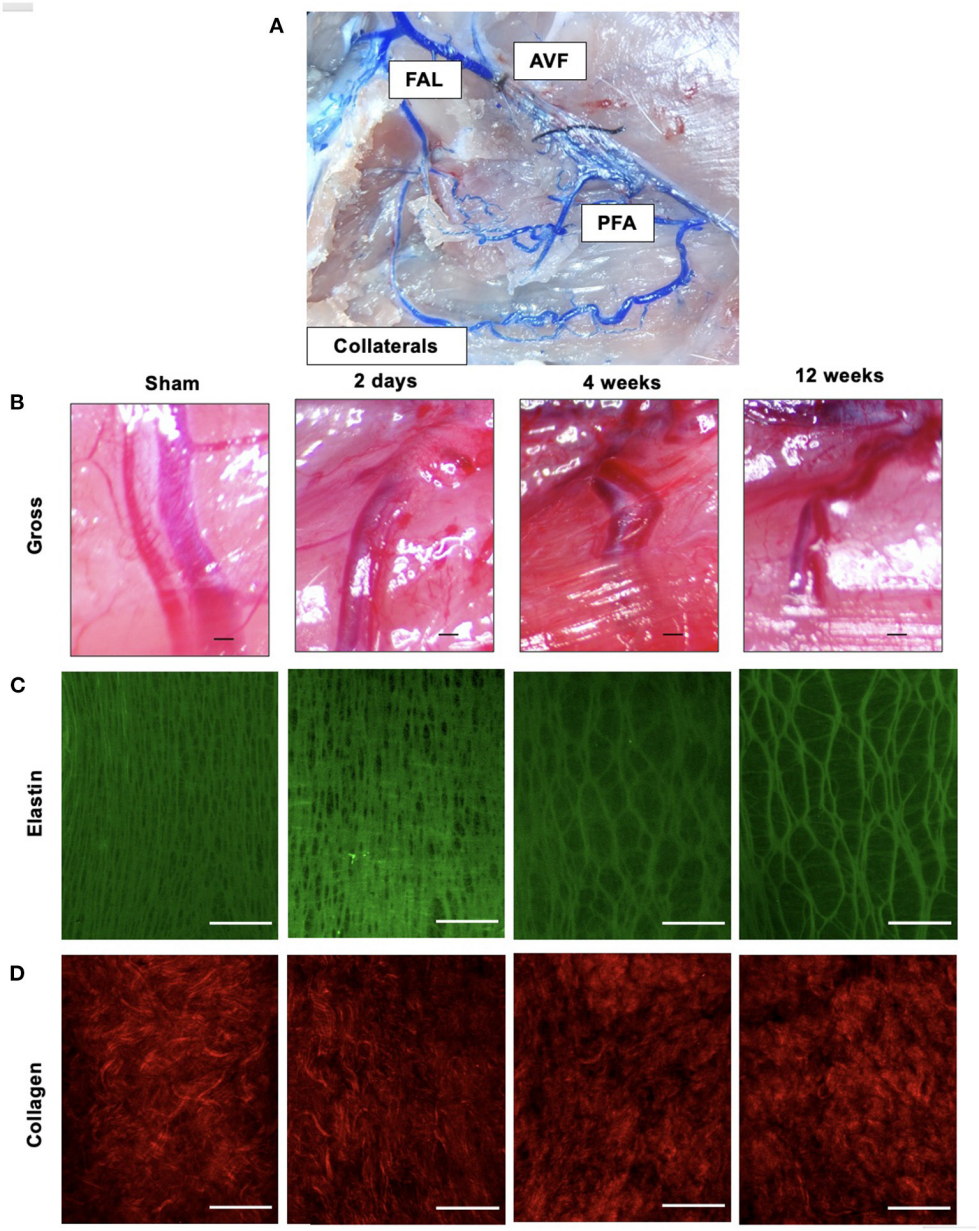
Outward remodeling occurs following FAL-AVF and results in destruction of the elastin framework within the IEL with preservation of collagen structure. **(A)** Microfil infused arterial tree within the FAL-AVF hindlimb. Labels delineate the relevant structures. **(B)** Gross light microscopy images of profunda arteries following sham operation and FAL-AVF at 2 days, 4 weeks, and 12 weeks. Scale bar = 500 μm. **(C)** Multiphoton images of the IEL following sham operation and FAL-AVF at 2 days, 4 weeks, and 12 weeks. Images acquired with Olympus FV1000MPE utilizing 830 nm laser. Elastin appears green. Scale bar = 50 μm. **(D)** Collagen appears red. Scale bar = 50 μm. FAL, femoral artery ligation; AVF, arteriovenous fistula; PFA, profunda femoris artery.

Gross images of the AVF and PFA collaterals were taken at time of sacrifice. Laser speckle contrast imaging (LSCI) was performed to confirm the function and success of the AVF procedure (Moor FLPI-2, Moor Instruments, Inc.). This was performed by evaluating the plantar paw in the experimental hindlimb and comparing the laser perfusion to the sham paw. Closure of the functioning AVF resulted in an increase in perfusion compared to sham (Supplementary Figure 1). To evaluate the arterial tree and adjacent structures more clearly, select animals at each time point were perfused with Microfil at time of sacrifice (1:1 mixture by body weight of compound to proprietary diluent and 5% curing agent; MV120, Flow Tech, Inc.). Once cured, the arterial tree and collaterals were dissected carefully and brightfield images were then captured (Figure 1A).

### Multiphoton Microscopy

Arterial segments were stored in 0.5% paraformaldehyde after fixation. In preparation for MPM, loose connective tissue and extraneous skeletal muscle were carefully removed and tissues mounted as described previously (14). MPM was performed using an Olympus FV 1,000 MPE microscope (Tokyo, Japan) with a Chameleon Ultra mode-locked Ti: Sapphire laser (Coherent, Palo Alto, CA) set to emit an 830 nm wavelength, through a 1.05 NA 25X water-immersion objective. The RXD1 channel (350–450 nm emission filter) allowed visualization of the fibrillar collagen via second harmonic generation, while the RXD2 channel (500–550 nm emission filter) was used to image the elastic fiber autofluorescence via two-photon excitation. Autofluorescence of elastin after two-photon excitation is displayed in green whereas collagen is displayed in red.

### mRNA Harvest and qPCR

The profunda femoris artery (PFA) was utilized for evaluation of collateral gene expression. At time of sacrifice, the artery was flushed with cold PBS by placing a catheter in the distal aorta. The PFA was then harvested from the tissue and snap frozen in liquid nitrogen. Samples were then stored at −80°C until use. Samples were then desiccated using a sterile bowl mortar and pestle, and further disrupted using Qiagen's QIAshredder and RNeasy kits to collect their RNA. The RNA samples were then converted into cDNA following Takara's RNA to cDNA EcoDry Premix (Oligo dT) kits, to be used for qPCR. The qPCR was carried out on Applied Biosystems QuantStudio 6 and 7 Real-Time PCR Systems, using their recommended reagents, plates and protocols. PCR runs were cycled between 95C and 60C 40 times. Primers used included: Fibrillin 1 (FBN-1), Fibrillin 2 (FBN-2), Lysyl oxidase 1 (LOX-1), Matrix Metalloproteinase 2 (MMP-2), Matrix Metalloproteinase 9 (MMP-9), Fibulin 4 (FBLN-4), and Fibulin 5 (FBLN-5), Tissue Inhibitor of Metalloproteinases 2 (TIMP-2), Tropoelastin, Cathepsin S (Cath-S) Cystatin C (Cys-C), Collagen 1 (Col-1), Collagen 3 (Col-3), Neutrophil Expressed Elastase (ELANE) and GAPDH. Relative gene expression was calculated using the ΔΔCq method. Expression of target genes was normalized to GAPDH mRNA level. All primers were pre-designed and validated primers for rats specifically and obtained from integrated DNA technologies (IDT; Coralville, Iowa). Primer sequences are listed in the Supplementary Table 1. *N* = 4–6 animals for each time point.

### Measuring Serum Desmosine

Whole blood was collected via cardiac puncture at time of sacrifice. The blood was allowed to coagulate at room temperature for 30 min and then centrifuged. Serum was collected and stored at −80°C until use. The desmosine concentration of serum samples were quantified using ELISA following the manufacturer's instructions (MyBiosource Rat Desmosine ELISA kit). N = 6-12 for each time point.

### Endothelial Flow Model

Commercially available human coronary artery endothelial cells (HCAEC) were obtained from the American Type Culture Collection (ATCC). Cells were cultured and transferred to channel slides for flow experiments. Experiments were performed using the ibidi Pump System (Ibidi; Martinsried, Germany) to create unidirectional flow to mimic physiological blood flow conditions. Shear stress is expressed in *dyn*·*s/cm*^2^. All cells were plated to confluence for flow experiments. Low shear stress was defined in this study as 8 *dyn*·*s/cm*^2^ and high shear stress was defined as 40 *dyn*·*s/cm*^2^. Cell populations were then conditioned with low shear for 24 h (control) with experimental groups undergoing and additional exposure to either 2, 4, or 6 h of high shear stress. These parameters were chosen based on prior physiologic studies that calculated aortic endothelial shear, which average from 3 to 6 *dyn*·*s/cm*^2^ in the pararenal aorta. In conditions of calculated collaterals or response to occlusions, the average range is 0.7–30 *dyn*·*s/cm*^2^ (15– 19). Cells were lysed and mRNA harvested using mRNEasy Kit per manufacturer instruction (Quiagen). PCR analysis was performed as described above.

Culture media from flow experiments was collected. Media was ultracentrifuged and cell pellet was resuspended in double distilled water. Each sample was standardized to contain 10,000 ng protein per milliliter using a bicinchoninic acid (BCA) protein assay kit (ThermoFischer BCA assay kit). Standardized samples were then evaluated for amount of MMP-2 and MMP-9 using commercial ELISA kits (biotechne Human total MMP-2 and MMP-9 ELISA kits). Cathepsin activity in the cell media was also evaluated using a commercial kit (abcam Cathepsin S Activity Assay Kit, fluorometric). Each assay was performed in triplicate and the average number is reported in the figures. *N* = 4 for each shear stress time point.

### Elastase Experiments

For intraluminal elastase exposure, the CFA was temporarily occluded proximal and distal to the superficial epigastric artery (SEA) branch. The SEA was cannulated with a microrenathane catheter (0.025″ diameter, Braintree Scientific). Porcine pancreatic elastase (PPE, 4–8 U/mL, 100–200 μl, Sigma-Aldrich) solution was instilled into the CFA and PFA branch through the catheter and allowed to dwell for 5 min prior to evacuation and irrigation with saline. Control arteries were instilled with saline alone. The catheter was removed, and the SEA was ligated. Flow was then restored into the CFA. Animals were sacrificed after 2 weeks and PFAs were harvested for microscopy analysis. *N* = 4 for both elastase and saline treated animals.

### Statistical Analysis

The mRNA relative fold-changes of target genes of experimental conditions relative to control for the PCR data were quantified using the 2^−ΔΔCq^ method. Comparisons between various time points after FAL-AVF and sham operations were made with non-parametric ANOVA analysis with Bonferroni adjustment. Results are expressed in figures and results as mean ± standard error of the mean (SEM). All calculations were performed in GraphPad statistical software suite.

## Results

### Elastic Fiber Continuity Over Time

Gross microscopy images of the arterial tree infused with Microfil after FAL + AVF are included in Figure 1A. Gross images of the profunda after FAL + AVF at various time points are shown in Figure 1B. The profunda progressively increases diameter and arterial tortuosity over time in response to FAL + AVF. Multiphoton microscopy (MPM) images at each time point are also displayed (Figures 1C,D). The IEL changed from a dense, elastic sheet with numerous small fenestrations to a more dispersed, web-like structure resulting from dramatic enlargement of fenestrations. Collagen is predominantly present in the adventitia, and did not demonstrate morphologic changes following FAL + AVF, consistent with our prior findings (13).

### Early Upregulation in Proteolytic Enzymes

**Figure 2:**
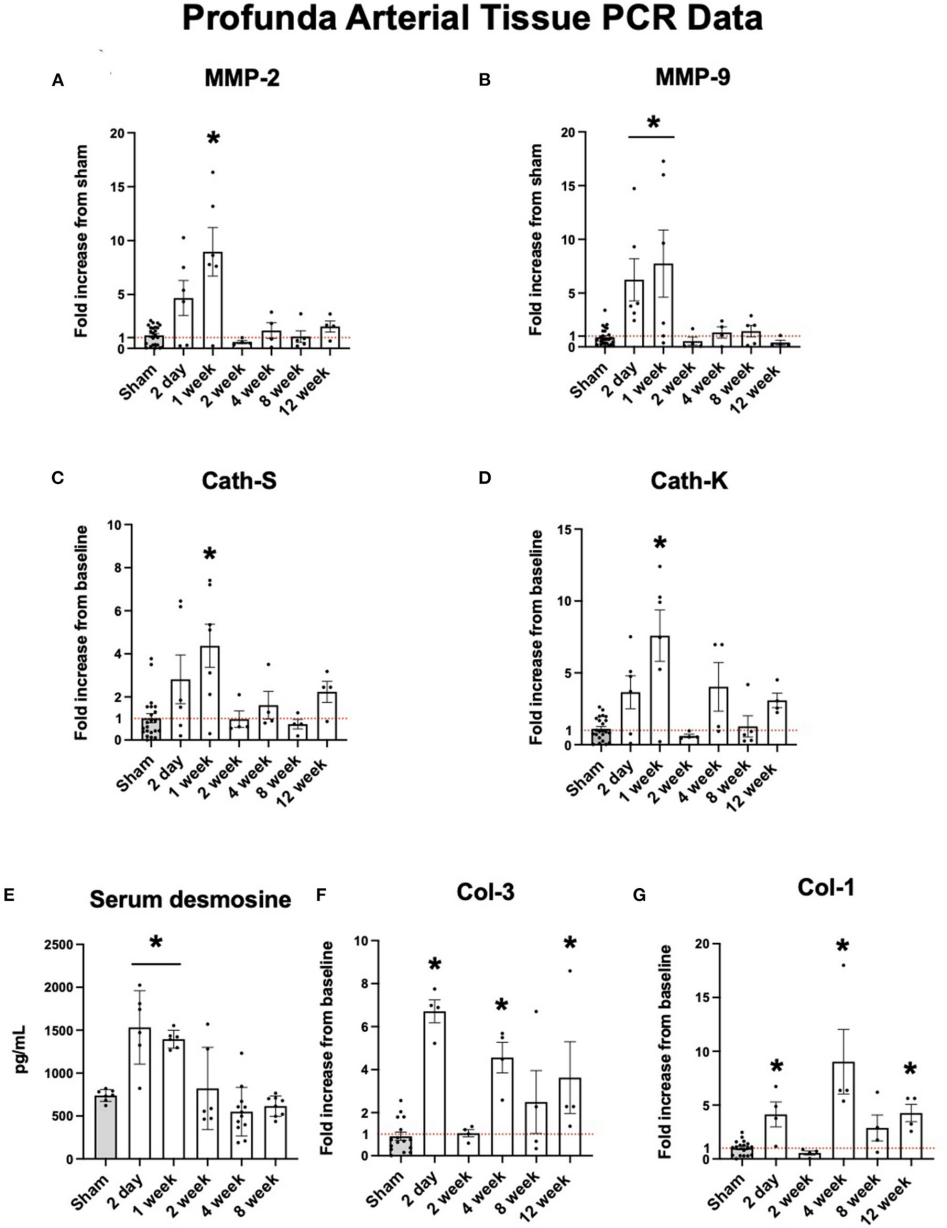
Upregulation of arterial elastolysis immediately following FAL-AVF that returns to baseline after one week. **(A)** PCR fold changes of the Matrix Metalloproteinase 2 (MMP-2) gene. **(B)** PCR fold changes of the Matrix Metalloproteinase 9 (MMP-9) gene. **(C)** PCR fold changes of the Cathepsin S (Cath-S) gene. **(D)** PCR fold changes of the Cathepsin K (Cath-K) gene. **(E)** Serum desmosine increases immediately after FAL-AVF and returns to baseline after one week. **(F)** PCR fold changes of the Collagen one (Col-1) gene. **(G)** PCR fold changes of the Collagen 3 (Col-3) gene. All time points compared to sham. *N* = 4–6 per timepoint. *p < 0.05. Red dotted line represents a fold change x1.

Proteolytic markers of elastolysis show similar trends. Elastolytic proteinases such as MMP-2 and MMP-9 are also increased early. At two days, the expression profiles of MMP-2 and MMP-9 were elevated nearly 10-fold before returning to baseline after one week (Figures 2A,B). Cathepsins S and K showed similar levels of expression (Figures 2C,D). The peak expression of elastolytic enzymes observed here coincided with serum levels of desmosine. By 2 days, mean serum desmosine reached a peak of 1,213 ± 670.8 pg/mL before returning to baseline levels as compared to sham operation by 2 weeks (Figure 2E).

### Collagen Proteins Remain Upregulated at Later Time Points

While elastolytic proteins showed early peaks in expression and later down regulation, which corresponds to what we see in the IEL structure with MPM microscopy, the same trends were not seen in collagen analyses. Both Col-1 and Col-3 showed increased expression after arterial occlusion at later time points (Figures 2F,G).

### Early Upregulation in Gene Expression of ECM Structural Proteins

**Figure 3:**
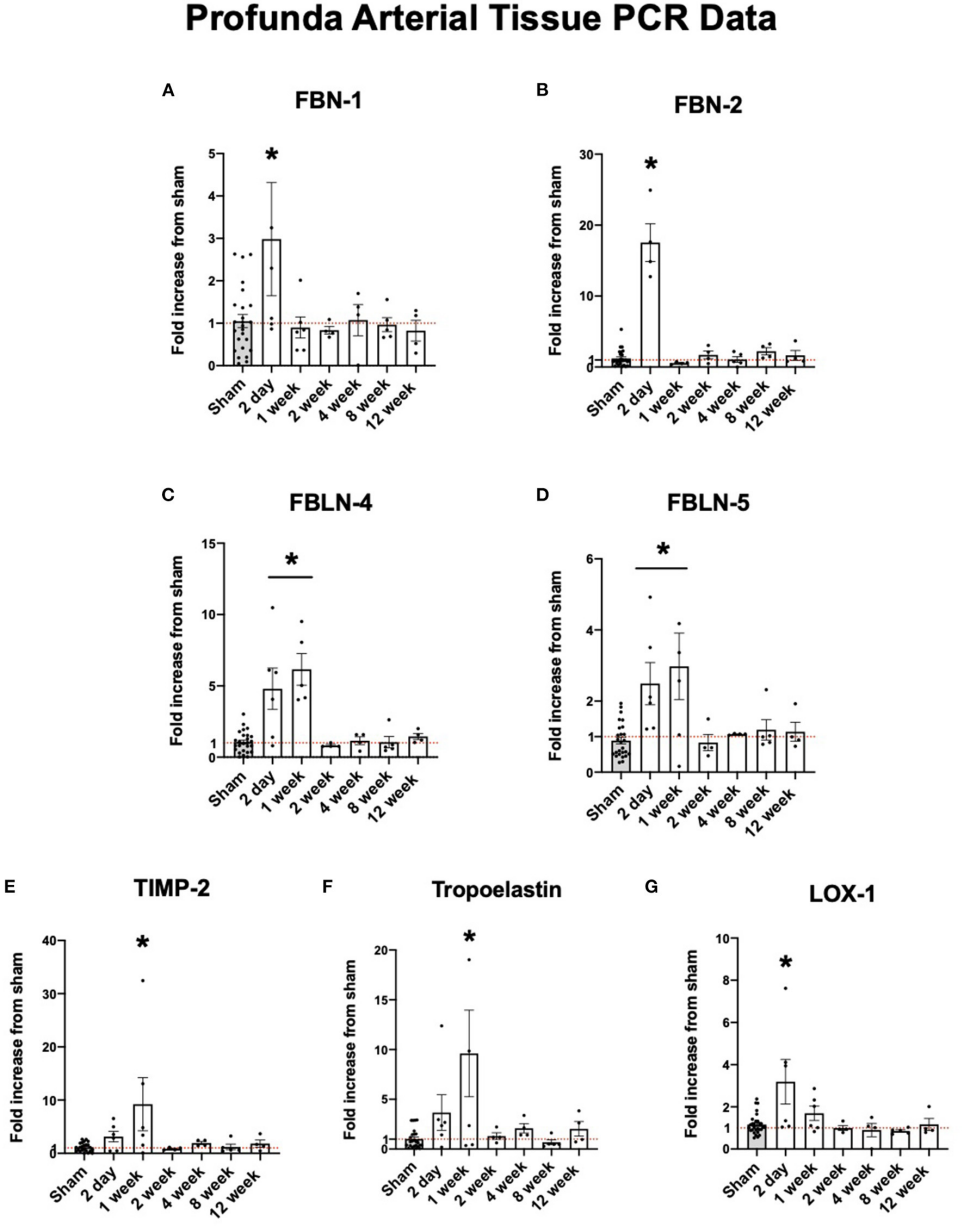
Upregulation of arterial elastogenesis immediately following FAL-AVF that returns to baseline after one week. **(A)** PCR fold changes of the Fibrillin 1 (FBN-1) gene. **(B)** PCR fold changes of the Fibrillin 2 (FBN-2) gene. **(C)** PCR fold changes of the Fibulin 4 (FBLN-4) gene. **(D)** PCR fold changes of the Fibulin 5 (FBLN-5) gene. **(E)** PCR fold changes of the Tissue inhibitor of metalloproteinases 2 (TIMP-2) gene. **(F)** PCR fold changes of the Tropoelastin gene. **(G)** PCR fold changes of the lysyl oxidase 1 (LOX-1) gene. All time points compared to sham. *N* = 4–6 per timepoint. *p < 0.05. Red dotted line represents a fold change x1.

Fibrillin-1 (FBN-1) and fibrillin-2 (FBN-2) provide support for elastic fiber integrity in the ECM (20). Fibulin-4 (FBLN-4) and fibulin-5 (FBLN-5) are described to be important in elastogenesis and arterial wall integrity (21). Lysyl oxidase (LOX-1) is an important protein in reinforcing and repairing elastic fibers as they are remodeled in arteriogenesis (22). Each of these structural proteins were initially upregulated in the collateral tissue before dropping down to sham levels of expression. As shown in Figures 3A–G, the expression of each of these proteins peak initially at 2 days to one week and fall to sham levels of expression after one week.

### HCAEC Response to Increased Shear Stress

**Figure 4:**
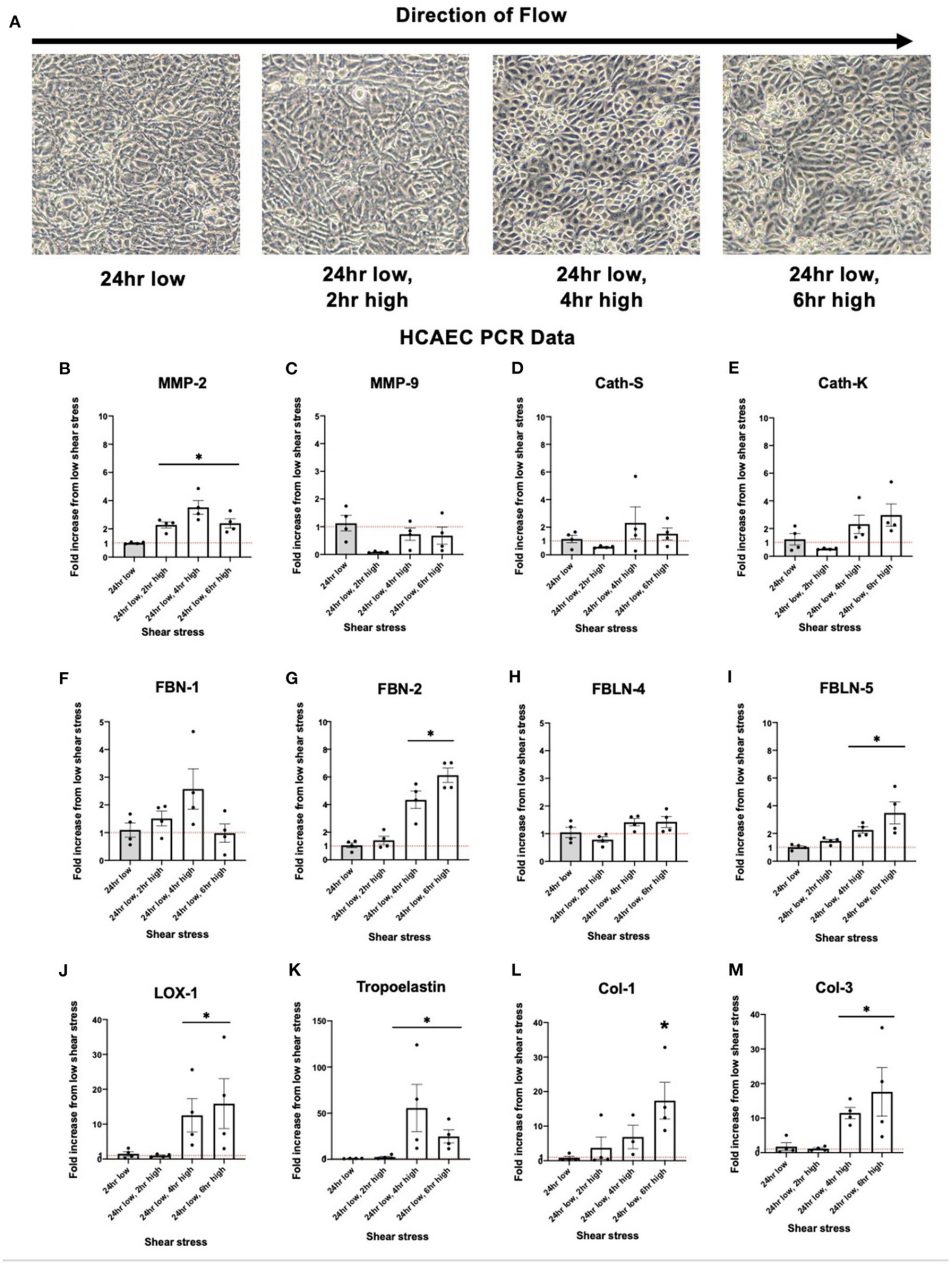
Upregulation of human coronary artery endothelial cell (HCAEC) elastogenesis and elastoloysis in response to increased shear stress displays similar patterns to rat arterial tissue. **(A)** Representative brightfield microscopy images of HCAEC confluence at each shear stress time point tested. **(B)** PCR fold changes of the MMP-2 gene. **(C)** PCR fold changes of the MMP-9 gene. **(D)** PCR fold changes of the Cath-S gene. **(E)** PCR fold changes of the Cath-K gene. **(F)** PCR fold changes of the FBN-1 gene. **(G)** PCR fold changes of the FBN-2 gene. **(H)** PCR fold changes of the FBLN-4 gene. **(I)** PCR fold changes of the FBLN-5 gene. **(J)** PCR fold changes of the LOX-1 gene. **(K)** PCR fold changes of the Tropoelastin gene. **(L)** PCR fold changes of the Col-1 gene. **(M)** PCR fold changes of the Col-3 gene. 24 h low shear stress conditions. *N* = 4 per timepoint. *p < 0.05. Red dotted line represents a fold change x1.

Brightfield images of HCAEC cells taken after exposure to varying levels of shear stress is displayed in Figure 4A. Expression of MMP-2 was significantly increased in response to increased shear stress. Other known proteins such as Cathepsin K, a potent elastin degradation protein (23, 24), and Cathepsin S, showed similar trends although the results were not statistically significant. Similar to collateral tissue, integral IEL structural proteins FBN-2, FBLN-5, and LOX-1 show an increase in expression with increased shear stress (Figures 4B–M).

**Figure 5:**
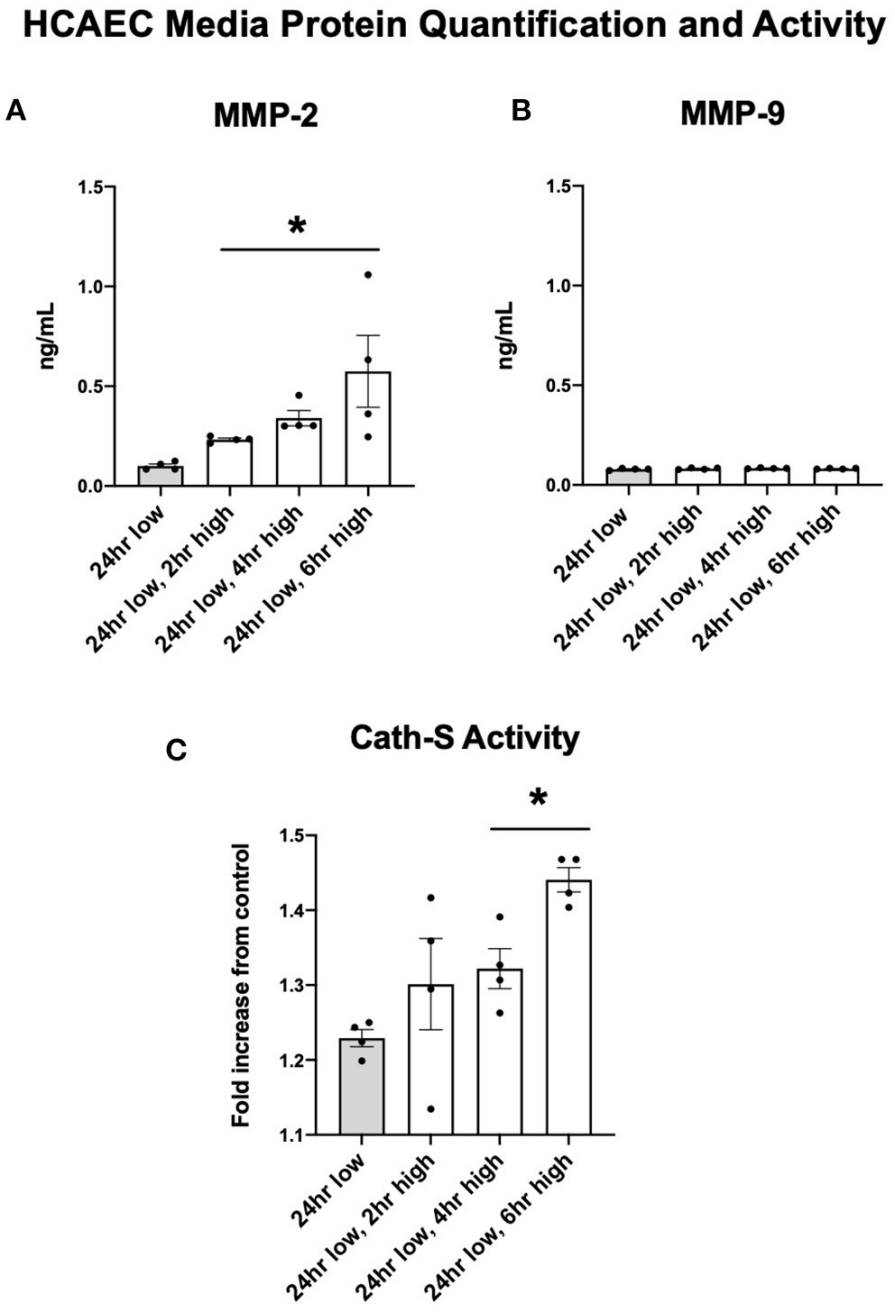
MMP-2 concentrations and Cathepsin activity increase with increases to shear stress while MMP-9 remains stable. **(A)** MMP-2 protein quantification in cell media after adjustments in shear stress. **(B)** MMP-9 protein quantification in cell media after adjustments in shear stress. **(C)** Cathepsin S enzymatic activity in cell media after adjustments in shear stress. All time points compared to 24 h low shear stress conditions. *N* = 4 per timepoint. *p < 0.05.

Protein quantification of MMP-2 in the cellular media reflected the upregulated expression seen in HCAEC. After six h of high shear stress, MMP-2 protein levels increased nearly five-fold (Figure 5A, *p* < 0.05). MMP-9 did not show the same discrepancy in protein levels and was in fact seen in very low concentrations regardless of shear stress exposure, suggesting that HCAECs produce very little MMP-9 (Figure 5B). Cathepsin S activity was increased with increased levels of shear stress at 4 and 6 h (Figure 5C).

### Elastase Degradation of IEL and Associated Matrix Changes

If elastin degradation is required for the restructuring of the IEL in arteriogenesis, we hypothesized that intraarterial elastase administration may produce qualitatively similar changes to the IEL even in the absence of an increased shear stress impetus for remodeling. PPE or saline was administered into isolated CFA and PFA segments via the SEA. After 2 weeks, saline treated arteries appeared grossly normal and retained a normal ECM morphology (Supplementary Figure 2) while PPE treated arteries demonstrated ~2-3-fold diameter expansion. For most of the PFAs (3/4) exposed to elastase, the IEL remained continuous with a loosened mesh-like appearance that resembled the FAL + AVF model.

## Discussion

We have previously found that during arteriogenesis, outward remodeling of the arterial wall required partial degradation of the IEL, but also required LOX for stabilization (13). We have also demonstrated that despite the large increases in vessel diameter, the mass of elastin within the vessel is essentially constant (13). In this study, we have shown upregulation of key elastolytic genes MMP-2, MMP-9, Cathepsin S, and Cathepsin K occur within the first week in a rat model of arteriogenesis. We found this corresponded with a spike in serum desmosine early in arteriogenesis, indicating the actual degradation of tissue elastin. Importantly, we found that ECM degradation appears to be simultaneously counterbalanced with local upregulation of elastic fiber components (tropoelastin, fibrillins and fibulins). However, our prior observation that no additional elastin mass is achieved among mature collateral arteries may indicate that only elastic fiber repair is occurring during arteriogenesis, without building of new elastic fibers. Indeed, inhibition of LOX activity with BAPN did not affect inhibit outward remodeling but did result in elastic fiber breaks in the IEL of developing collaterals, and eventual loss of IEL entirely (13).

Human elastin production occurs primarily in development, and it is observed that essentially no new elastin is produced in mature tissues (25, 26). Ninety percent of elastic fiber mass is composed of elastin itself, with the remainder fibrillar proteins and proteoglycans oriented at the periphery of the fiber. During development, tropoelastin monomers (produced by resident vascular cells) self-assemble in the extracellularly and associate with a microfibril scaffold before undergoing crosslinking reactions mediated by LOX (27). Notably, elastin is the longest-lasting protein in mammals with an estimated half-life approaching the human life span (28). In contrast, collagens have half-lives on the order of two weeks under some conditions, necessitating continual synthesis for replacement and reinforcement (29, 30).

Arterial structure is reliant on ECM integrity (31). Elastin fibers are essential to the stretch and recoil needed in the arterial ECM (32). Elastin fibers interact with collagen to create a framework to store energy and uniformly distribute stress throughout the arterial wall. Previous studies have suggested that vertebrates rely on a limited quantity of elastic fibers throughout their lifetime, making them susceptible to age and disease related degeneration over time (33). This suggests that any type of elastolysis would affect structural integrity with a lack of elastogenesis. Previous reports have found that while elastic fibers can be repaired, they cannot be replaced once destroyed (34, 35).

There are a variety of proteins involved in ECM structure and integrity. Here, we investigated several proteins involved in both stability and degradation of the ECM. Elastin is a key component of the ECM and is primarily composed of elastic fibers. Elastic fibers interact with fibrillar proteins and proteoglycans to create the elastin structure. Elastic fibers start to disassemble during early arteriogenesis. Proteolytic enzymes such as MMP-2 and MMP-9 have been implicated in collateral formation and growth (10, 36). In whole arterial tissue, we saw an early increase in gene expression for MMP-2 and−9 after arterial occlusion. This correlates to the similar early peak in circulating elastin-specific degradation product, desmosine. Upregulation of these proteins is as high as 10 times compared to the sham operated hindlimbs. This suggests that there is initially increased proteolysis and elastolysis. Both desmosine and MMP-2 and−9 levels drop after one week, suggesting decreased elastic fiber break down. In isolated endothelial cells, there is no significant increase in MMP-2 expression however MMP-2 is significantly downregulated after prolonged exposure to shear stress. This suggests that flow-responsive endothelial cells may be integral in the control of elastolysis.

Fibrillins and fibulins are integral in elastic fiber structure assembly and support (27, 37). Here, we see an early increase in gene expression for fibrillins-1 and−2 as well as fibulins-4 and−5 after arterial occlusion, which corresponds to increase in proteolysis. This suggests there is a significant elevation in elastic fiber destruction and repair early after arterial occlusion. At two days, these support proteins peak in expression, while elastic fiber integrity remains intact. However, after this time threshold, these structural proteins are no longer upregulated compared to sham operated hindlimbs. This suggests destruction of the elastic fibers with no further elastogenesis to promote the use of these structural proteins. Conversely, collagen related proteins do not change significantly in their expression, rather they are upregulated at later time points in response to arterial occlusion. This is consistent with what we see in microscopy, in that collagen integrity in the adventitia is retained, unlike the IEL elastin fiber integrity. In isolated endothelial cells, low shear stress to high shear stress causes activation of a similar response. FBN-1 and FBLN-4 are initially upregulated in response to a change in shear stress however their expression rapidly decreases after prolonged high shear conditions, suggesting mechanoreceptive endothelial cells are integral in the initial response to flow changes but are likely less important after prolonged exposure to increased shear stress. It is likely that the endothelium is the site of mechanotransduction and initiation of arteriogenesis through elastic fiber regulation of these specific proteins. Interestingly, for most of the PFAs exposed to elastase, the IEL remained continuous with a loosened mesh-like appearance that resembled the FAL+AVF model. This similar pattern of IEL change in response to uninhibited elastolysis suggests similarities between the two models and the importance of the balance between elastogenesis and elastolysis in arterial remodeling.

Human coronary endothelial cells displayed similar patterns to altered shear stress as rat profunda arterial tissue. Indeed, electrolytic protein MMP-2 exhibited increased expression with response to increased shear stress. This corresponded to an increase in protein detected in cellular media. Elastogenic proteins FBN-2, FBLN-5, LOX-1, and tropoelastin were all upregulated in a similar manner. This could reflect the same processes observed after an increase in shear stress exhibited in collateral vessels in response to arterial occlusion. The patterns are not exactly replicated from the *in vivo* studies, likely because these processes are not entirely isolated to the arterial endothelium.

This study has several limitations. First, we observe various trends in the expression of key regulators of structural ECM remodeling in both a rat tissue and human endothelial cells in models of arteriogenesis. However, we have not directly confirmed the activity of these elastolytic enzymes during outward remodeling, as they are produced as pro-enzymes that when activated, are further subject to endogenous inhibitors. Second, our rodent model of arteriogenesis using FAL + AVF may not accurately represent the human mechanics of arteriogenesis, although similar models have long been used to approximate adaptive responses to AODs. The current study was also not aimed at identifying specific tissue cell types that are responsible for the gene expression that we observed. During arteriogenesis, a diverse population of resident arterial wall cells as well as a changing panel of occupying inflammatory cells contribute to the remodeling process. However, endothelial cell activation is likely responsible for initiation of arteriogenesis, and the expression of elastolytic agents in response to elevated shear stress may indicate the endothelium as an important source of IEL degradation. Finally, while our study largely focused on elastic fiber modifications, we recognize that collagen and numerous other ECM components have important structural roles and are likely involved as well. Further study of the mechanisms of IEL remodeling in outward arterial remodeling is warranted.

## Conclusion

Arterial occlusive disease is a highly prevalent problem. Novel strategies to enhance arteriogenesis and increase collateral effectiveness would be clinically useful in this patient population. Elastin degradation is a key feature of arteriogenesis initiation and is likely mediated by local upregulation of elastolytic genes. We have demonstrated that simultaneous production of elastolytic factors and components of elastic fiber repair occurs in the IEL during periods of arterial expansion. Arterial endothelial cells may be critical in the initiation of IEL remodeling and begin ECM remodeling in response to altered shear stress.

## Data Availability Statement

The original contributions presented in the study are included in the article/Supplementary Materials, further inquiries can be directed to the corresponding author/s.

## Ethics Statement

The animal study was reviewed and approved by Institutional Animal Care and Use Committee at the University of Pittsburgh (IACUC Protocol #19095696).

## Author Contributions

Conceptualization and design of the study were performed by RM and EA. EA, NS, DM, and RK performed experimental and statistical analyses. RM, EA, and ET performed interpretation and curation of the data. Original draft written by EA. RM, DM, and NS wrote sections of the manuscript. All authors contributed to the article and approved the submitted version.

## Funding

This project was funded in part by the Vascular Cures Foundation and US Veterans Affairs (No. IK2BX003509 to RM) and National Heart, Lung, and Blood Institute (No. T32HL098036 to EA and RK). The University of Pittsburgh holds a Physician-Scientist Institutional Award from the Burroughs Wellcome Fund to EA.

## Conflict of Interest

The authors declare that the research was conducted in the absence of any commercial or financial relationships that could be construed as a potential conflict of interest.

## Publisher's Note

All claims expressed in this article are solely those of the authors and do not necessarily represent those of their affiliated organizations, or those of the publisher, the editors and the reviewers. Any product that may be evaluated in this article, or claim that may be made by its manufacturer, is not guaranteed or endorsed by the publisher.

## Supplementary Material

Supplementary Figure 1 Laser doppler images were used to test the function of the arteriovenous fistula. An increase in perfusion is seen when the fistula is occluded compared to the sham limb.

Supplementary Figure 2 FAL-AVF treatment results in IEL degradation, similar to elastase treatment. Representative multiphoton microscopic images taken from arterial tissue two weeks after exposure to either saline perfusion or elastase (PPE) perfusion. Images acquired with Olympus FV1000MPE utilizing 830 nm laser. Elastin appears green. *N* = 4. Bar = 50 μm.

Supplementary Table 1 Primer sequences of each primer used.

## References

[1] Criqui MH Matsushita K Aboyans V Hess CN Hicks CW Kwan TW. Lower extremity peripheral artery disease: contemporary epidemiology, management gaps, and future directions: a scientific statement from the american heart association. Circulation. (2021). 144:e171–e191. 10.1161/CIR.0000000000001005 34315230 PMC9847212

[2] Gerhard-Herman MD Gornik HL Barrett C Barshes NR Corriere MA Drachman DE. 2016 AHA/ACC guideline on the management of patients with lower extremity peripheral artery disease: executive summary. Vasc Med. (2017) 22:NP1–43. 10.1016/j.jacc.2016.11.008 28494710

[3] Berry C Balachandran KP L'Allier PL Lespérance J Bonan R Oldroyd KG. Importance of collateral circulation in coronary heart disease. Eur Heart J. (2007) 28:278–91. 10.1093/eurheartj/ehl446 17218452

[4] Meier P Gloekler S Zbinden R Beckh S de Marchi SF Zbinden S. Beneficial effect of recruitable collaterals: a 10-year follow-up study in patients with stable coronary artery disease undergoing quantitative collateral measurements. Circulation. (2007) 116:975–83. 10.1161/CIRCULATIONAHA.107.703959 17679611

[5] Meier P Hemingway H Lansky AJ Knapp G Pitt B Seiler C. The impact of the coronary collateral circulation on mortality: a meta-analysis. Eur Heart J. (2012) 33:614–21. 10.1093/eurheartj/ehr308 21969521

[6] Yang B-L Wu S Wu X Li MB Zhu W Guan Y. Effect of shunting of collateral flow into the venous system on arteriogenesis and angiogenesis in rabbit hind limb. Acta Histochem Cytochem. (2013) 46:1–10. 10.1267/ahc.12025 23554534 PMC3596601

[7] McEnaney RM McCreary D Tzeng E. A modified rat model of hindlimb ischemia for augmentation and functional measurement of arteriogenesis. J Biol methods. (2018) 5:e89. 10.14440/jbm.2018.234 31435496 PMC6703558

[8] Buschmann I Heil M Jost M Schaper W. Influence of inflammatory cytokines on arteriogenesis. Microcirculation. (2003) 10:371–9. 10.1080/mic.10.3-4.371.379 12851653

[9] Heil M Ziegelhoeffer T Wagner S Fernández B Helisch A Martin S. Collateral artery growth (arteriogenesis) after experimental arterial occlusion is impaired in mice lacking CC-chemokine receptor-2. Circ Res. (2004) 94:671–7. 10.1161/01.RES.0000122041.73808.B5 14963007

[10] Dodd T Jadhav R Wiggins L Stewart J Smith E Russell JC. MMPs 2 and 9 are essential for coronary collateral growth and are prominently regulated by p38 MAPK. J Mol Cell Cardiol. (2011) 51:1015–25. 10.1016/j.yjmcc.2011.08.012 21884701 PMC3208797

[11] Cai W Vosschulte R Afsah-Hedjri A Koltai S Kocsis E Scholz D. Altered balance between extracellular proteolysis and antiproteolysis is associated with adaptive coronary arteriogenesis. J Mol Cell Cardiol. (2000) 32:997–1011. 10.1006/jmcc.2000.1137 10888253

[12] Heil M Schaper W. Insights into pathways of arteriogenesis. Curr Pharm Biotechnol. (2007) 8:35–42. 10.2174/138920107779941408 17311551

[13] McEnaney R McCreary DD Skirtich NO Andraska E Sachdev U TE. Elastic laminar reorganization occurs with outward diameter expansion during collateral artery growth and requires lysyl oxidase for stabilization. Preprints. (2021) 11:7. 10.3390/cells11010007 PMC8750335 35011567

[14] McCreary DD Skirtich NO Andraska EA Tzeng E MR. Survey of the extracellular matrix architecture across the rat arterial tree. JVS Vasc Sci. (2021). 10.1016/j.jvssci.2021.08.001 PMC8739875 35028599

[15] Guo Z-Y Yan Z-Q Bai L Zhang M-L Jiang Z-L. Flow shear stress affects macromolecular accumulation through modulation of internal elastic lamina fenestrae. Clin Biomech. (2008) 23(Suppl. 1):S104–11. 10.1016/j.clinbiomech.2007.08.017 17923177

[16] Platt MO Ankeny RF Shi G-P Weiss D Vega JD Taylor WR. Expression of cathepsin K is regulated by shear stress in cultured endothelial cells and is increased in endothelium in human atherosclerosis. Am J Physiol Heart Circ Physiol. (2007) 292:H1479–86. 10.1152/ajpheart.00954.2006 17098827

[17] Ballermann BJ Dardik A Eng E Liu A. Shear stress and the endothelium. Kidney Int Suppl. (1998) 67:S100–8. 10.1046/j.1523-1755.1998.06720.x 9736263

[18] Hennig T Mogensen C Kirsch J Pohl U Gloe T. Shear stress induces the release of an endothelial elastase: role in integrin α(v)β(3)-mediated FGF-2 release. J Vasc Res. (2011) 48:453–64. 10.1159/000327009 21691119

[19] Reneman RS Hoeks APG. Wall shear stress as measured *in vivo*: consequences for the design of the arterial system. Med Biol Eng Comput. (2008) 46:499–507. 10.1007/s11517-008-0330-2 18324431 PMC2441533

[20] Whiteman P Hutchinson S Handford PA. Fibrillin-1 misfolding and disease. Antioxid Redox Signal. (2006) 8:338–46. 10.1089/ars.2006.8.338 16677079

[21] Yamashiro Y Papke CL Kim J Ringuette L-J Zhang Q-J Liu Z-P. Abnormal mechanosensing and cofilin activation promote the progression of ascending aortic aneurysms in mice. Sci Signal. (2015) 8:ra105. 10.1126/scisignal.aab3141 26486174 PMC5572214

[22] Chow M-J. Mechanics and Mechanobiology of Arteries: Contributions and Interactions of Collagen and Elastin. ProQuest Dissertations and Theses. Ann Arbor: Boston University (2013).

[23] Lai C-H Chang J-Y Wang K-C Lee F-T Wu H-L Cheng T-L. Pharmacological inhibition of cathepsin s suppresses abdominal aortic aneurysm in mice. Eur J Vasc Endovasc Surg Off J Eur Soc Vasc Surg. (2020) 59:990–9. 10.1016/j.ejvs.2020.01.008 32033870

[24] Panwar P Hedtke T Heinz A Andrault P-M Hoehenwarter W Granville DJ. Expression of elastolytic cathepsins in human skin and their involvement in age-dependent elastin degradation. Biochim Biophys Acta Gen Subj. (2020) 1864:129544. 10.1016/j.bbagen.2020.129544 32007579

[25] Wagenseil JE Mecham RP. Vascular extracellular matrix and arterial mechanics. Physiol Rev. (2009) 89:957–89. 10.1152/physrev.00041.2008 19584318 PMC2775470

[26] Wagenseil JE Mecham RP. New insights into elastic fiber assembly. Birth Defects Res C Embryo Today. (2007) 81:229–40. 10.1002/bdrc.20111 18228265

[27] Kozel BA Mecham RP. Elastic fiber ultrastructure and assembly. Matrix Biol. (2019) 84:31–40. 10.1016/j.matbio.2019.10.002 31669522 PMC8409341

[28] Shapiro SD Endicott SK Province MA Pierce JA Campbell EJ. Marked longevity of human lung parenchymal elastic fibers deduced from prevalence of D-aspartate and nuclear weapons-related radiocarbon. J Clin Invest. (1991) 87:1828–34. 10.1172/JCI115204 2022748 PMC295305

[29] Ooshima A Fuller GC Cardinale GJ Spector S Udenfriend S. Increased collagen synthesis in blood vessels of hypertensive rats and its reversal by antihypertensive agents. Proc Natl Acad Sci USA. (1974) 71:3019–23. 10.1073/pnas.71.8.3019 4370097 PMC388611

[30] Nissen R Cardinale GJ Udenfriend S. Increased turnover of arterial collagen in hypertensive rats. Proc Natl Acad Sci USA. (1978) 75:451–3. 10.1073/pnas.75.1.451 272662 PMC411267

[31] Ushiki T. Collagen fibers, reticular fibers and elastic fibers. A comprehensive understanding from a morphological viewpoint. Arch Histol Cytol. (2002) 65:109–26. 10.1679/aohc.65.109 12164335

[32] Wolinsky H Glagov S. Structural basis for the static mechanical properties of the aortic media. Circ Res. (1964) 14:400–13. 10.1161/01.RES.14.5.400 14156860

[33] Baldwin AK Simpson A Steer R Cain SA Kielty CM. Elastic fibres in health and disease. Expert Rev Mol Med. (2013) 15:e8. 10.1017/erm.2013.9 23962539

[34] Alves C Araújo AD Oliveira CLN Imsirovic J Bartolák-Suki E Andrade JS. Homeostatic maintenance via degradation and repair of elastic fibers under tension. Sci Rep. (2016) 6:27474. 10.1038/srep27474 27279029 PMC4899696

[35] Stone PJ Morris SM Martin BM McMahon MP Faris B Franzblau C. Repair of protease-damaged elastin in neonatal rat aortic smooth muscle cell cultures. J Clin Invest. (1988) 82:1644–54. 10.1172/JCI113776 3141480 PMC442733

[36] Dodd T Wiggins L Hutcheson R Smith E Musiyenko A Hysell B. Impaired coronary collateral growth in the metabolic syndrome is in part mediated by matrix metalloproteinase 12-dependent production of endostatin and angiostatin. Arterioscler Thromb Vasc Biol. (2013) 33:1339–49. 10.1161/ATVBAHA.113.301533 23599440 PMC3753795

[37] Walker-Caprioglio HM Trotter JA Little SA McGuffee LJ. Organization of cells and extracellular matrix in mesenteric arteries of spontaneously hypertensive rats. Cell Tissue Res. (1992) 269:141–9. 10.1007/BF00384734 1423476

