# Supplementary figures and images for: Simultaneous Upregulation of Elastolytic and Elastogenic Factors Are Necessary for Regulated Collateral Diameter Expansion
Source: Front Cardiovasc Med. 2022 Jan 12;8:762094. doi: 10.3389/fcvm.2021.762094 (PMC8789883; doi:10.3389/fcvm.2021.762094)

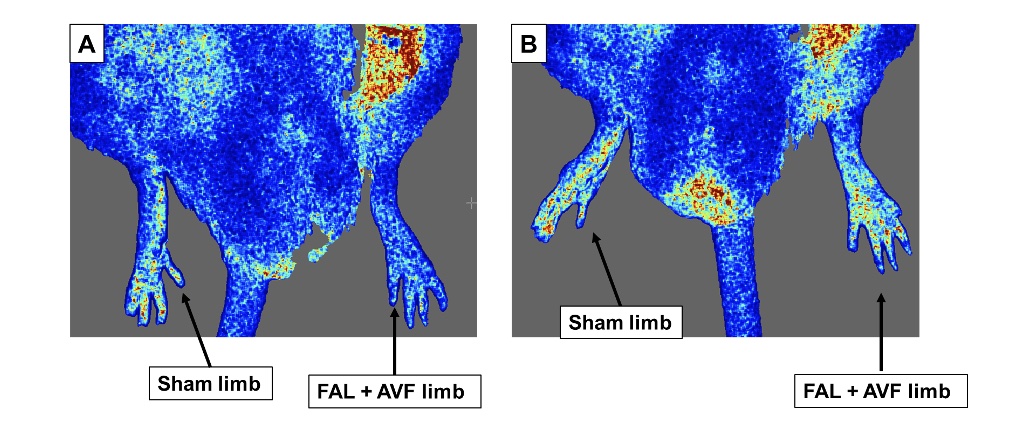

Supplement: Supplementary Figure 1 — Laser doppler images were used to test the function of the arteriovenous fistula. An increase in perfusion is seen when the fistula is occluded compared to the sham limb. [file Image_1.JPEG]

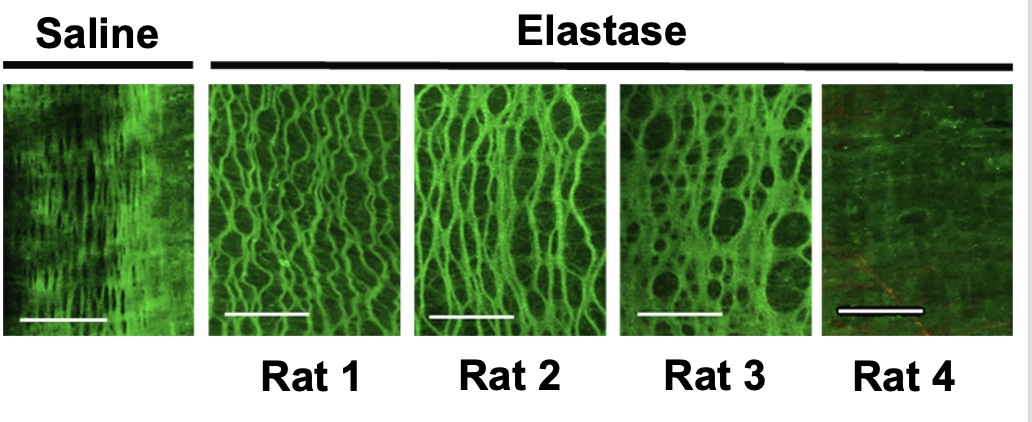

Supplement: Supplementary Figure 2 — FAL-AVF treatment results in IEL degradation, similar to elastase treatment. Representative multiphoton microscopic images taken from arterial tissue two weeks after exposure to either saline perfusion or elastase (PPE) perfusion. Images acquired with Olympus FV1000MPE utilizing 830 nm laser. Elastin appears green. N = 4. Bar = 50 μm. [file Image_2.JPEG]

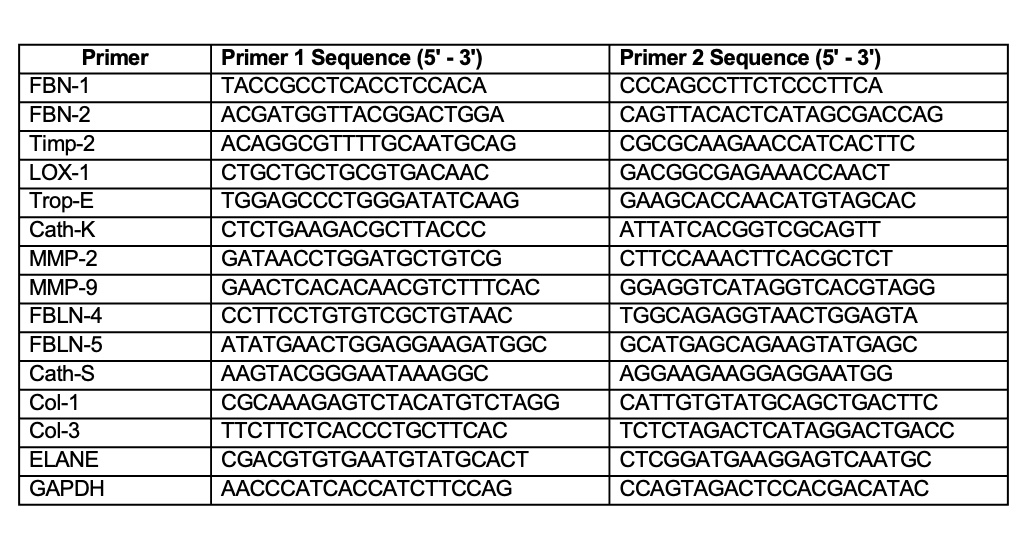

Supplement: Supplementary Table 1 — Primer sequences of each primer used. [file Image_3.JPEG]
