# Supplementary material for: Simultaneous Upregulation of Elastolytic and Elastogenic Factors Are Necessary for Regulated Collateral Diameter Expansion
Source: Front Cardiovasc Med. 2022 Jan 12;8:762094. doi: 10.3389/fcvm.2021.762094 (PMC8789883; doi:10.3389/fcvm.2021.762094)

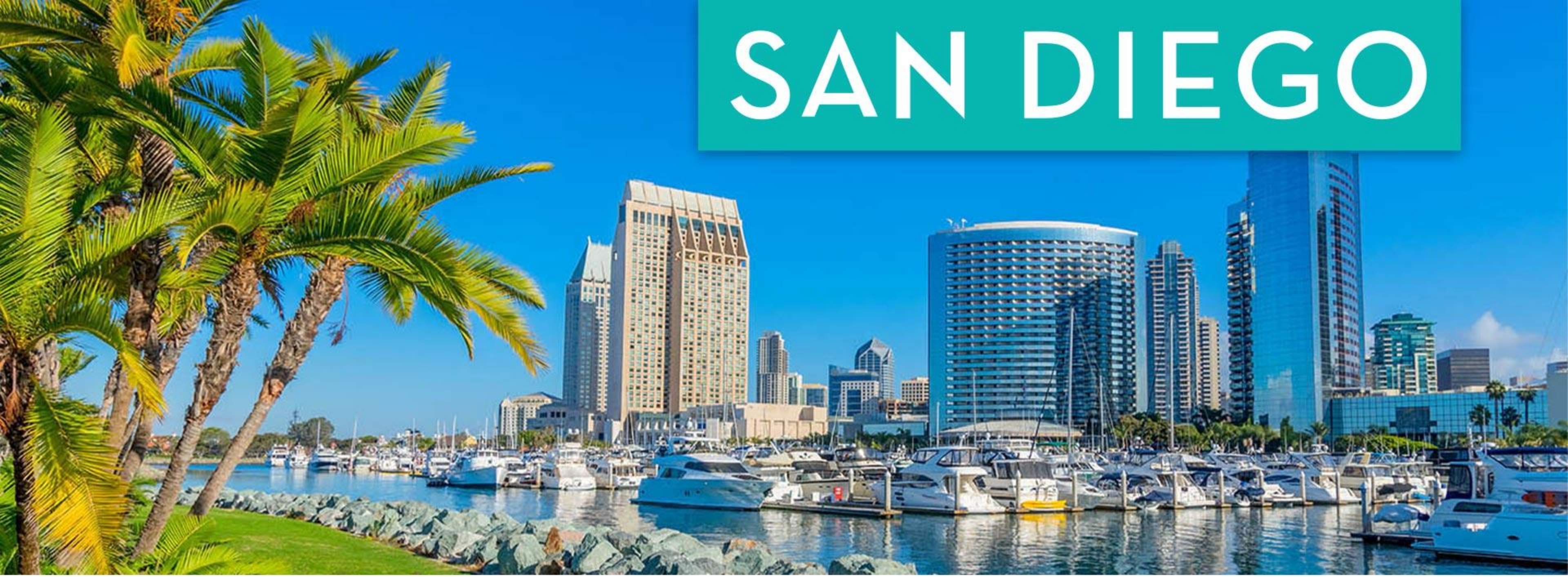


# 2021 VASCULAR ANNUAL MEETING®

# Simultaneous upregulation of elastolytic and elastogenic factors are necessary for regulated collateral diameter expansion

Andraska EA[1], Skirtich N[2], McCreary D[2], Kulkarni R[1], Tzeng E[1], McEnaney R[1]
[1]University of Pittsburgh Medical Center, Division of Vascular Surgery
[2]University of Pittsburgh School of Medicine

**PRESENTED BY:**

**Elizabeth Andraska, MD**
University of Pittsburgh, Vascular Surgery Resident

# Disclosure

We have no disclosures

# Arteriogenesis

- Collateral arteries develop through a process known as arteriogenesis
- Response to changes in fluid shear stress after conductance arterial occlusion
- Collateral arteries are important in maintaining perfusion of tissues
- Collaterals are not typically sufficient to fully replace the occluded artery

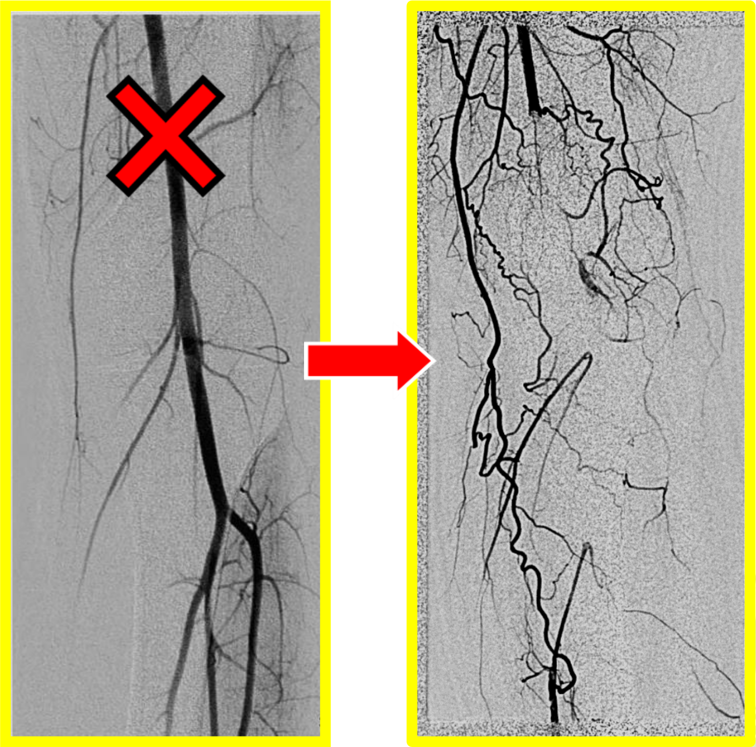

# Baseline arterial structure

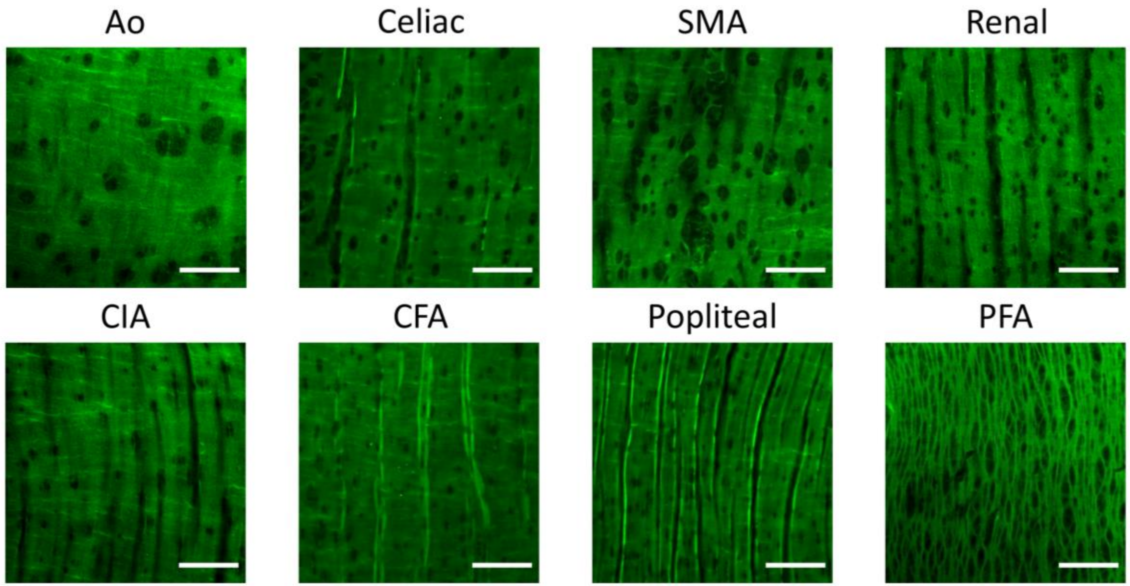


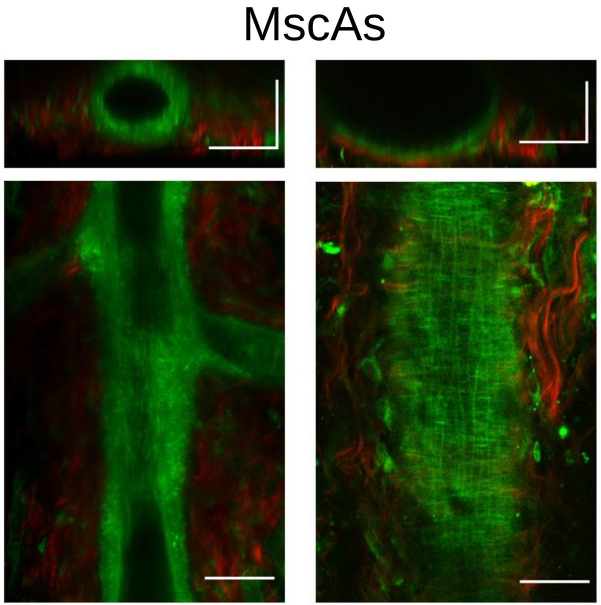


Multiphoton microscopic images of the internal elastic lamina (green autofluorescence of elastin by two-photon excitation)

# Starting point regarding IEL elastic content and morphology

PFA

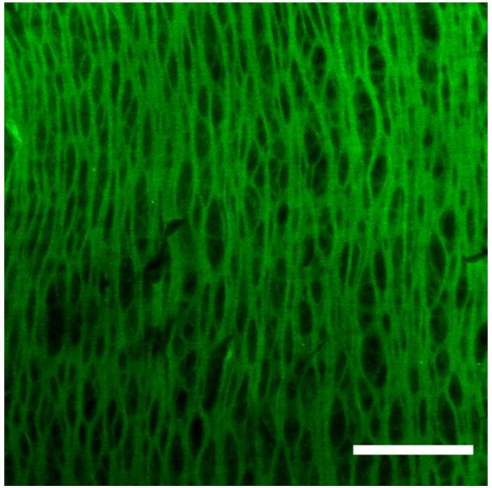

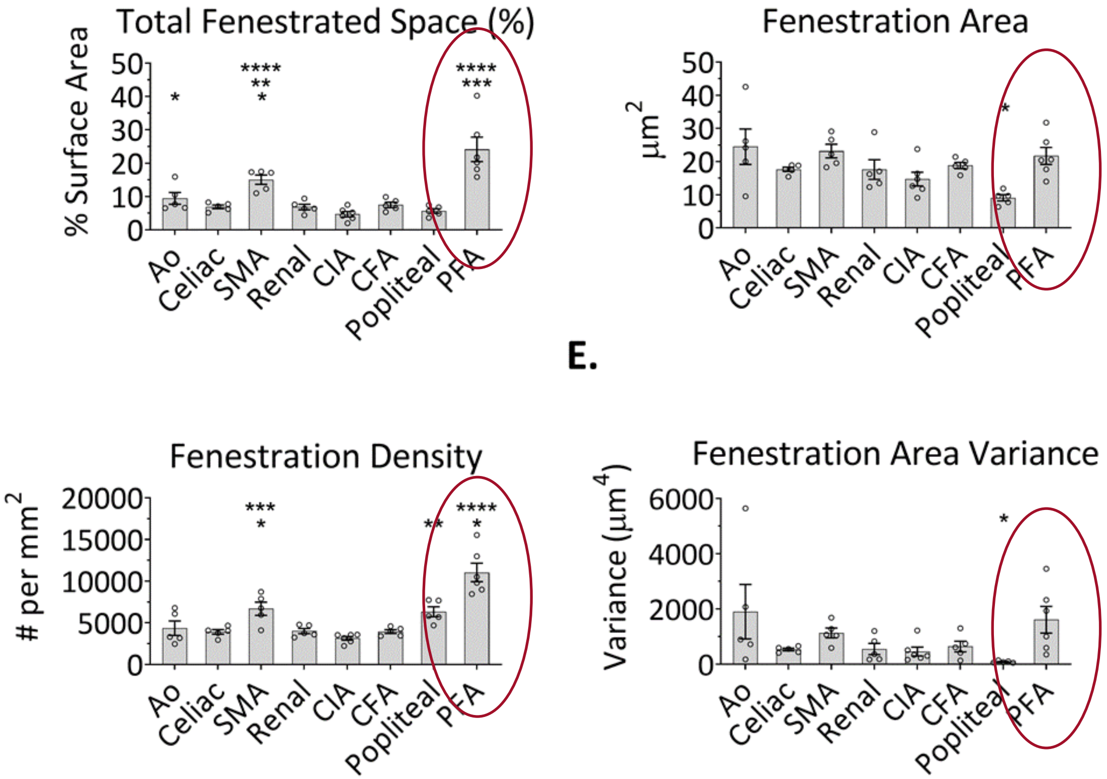

# Model: Femoral Artery Ligation + AV Fistula Formation

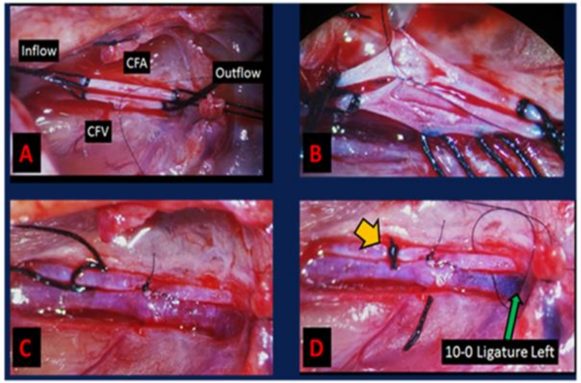


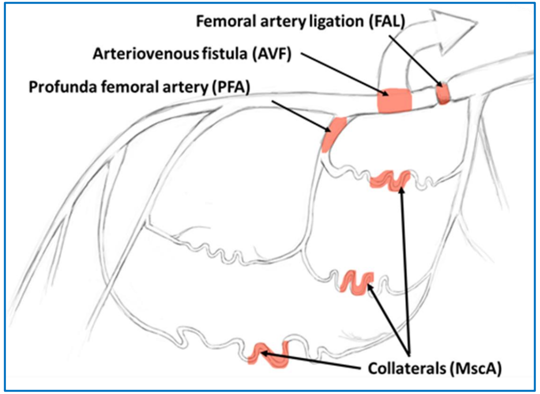


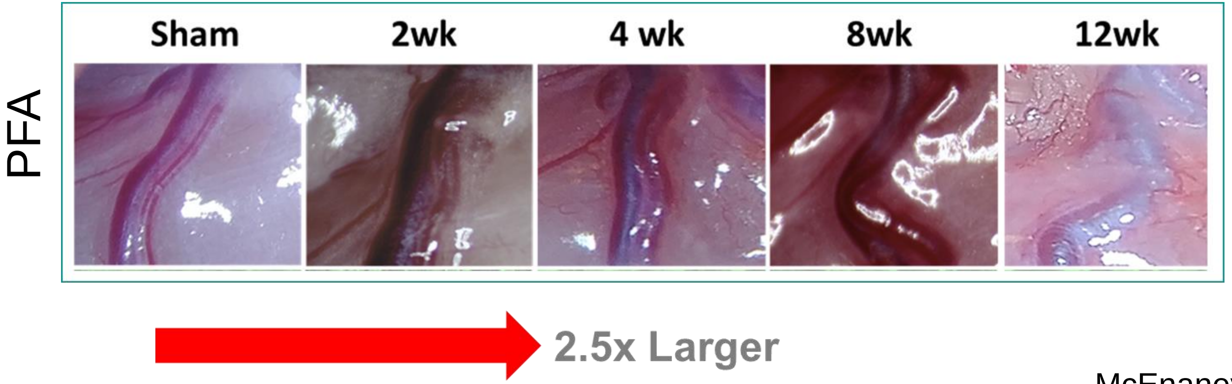


McEnaney et al. J Biol Methods 2018

# Structural changes with arteriogenesis

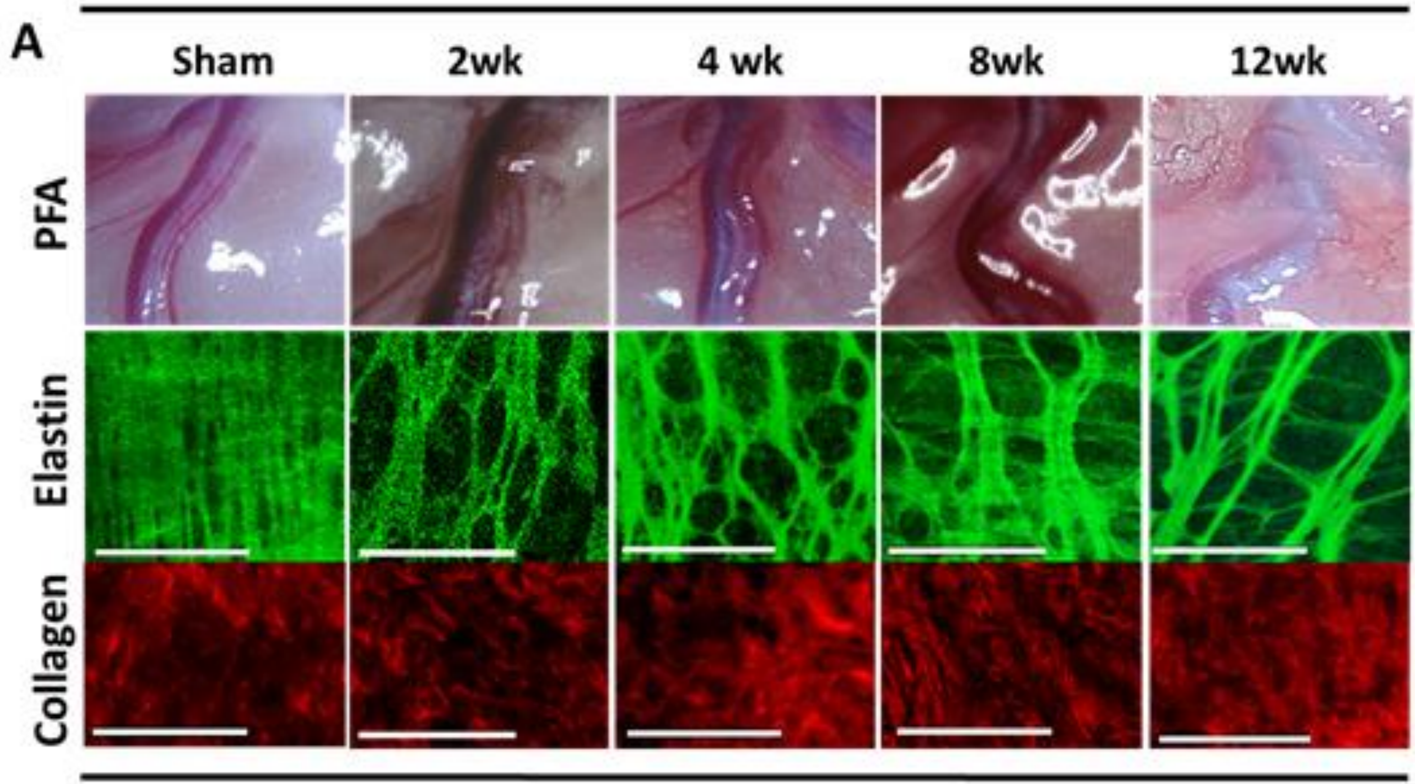


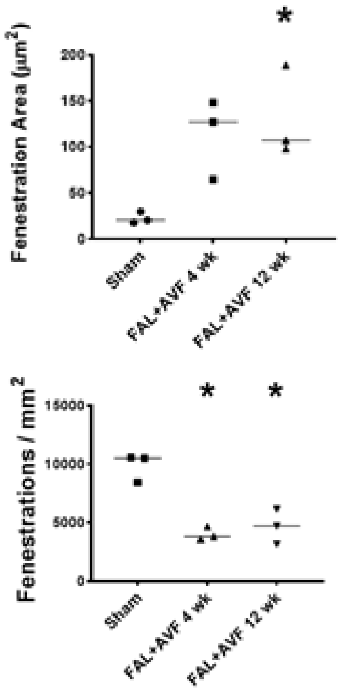


Multiphoton images of elastin (2 photon excitation) and collagen (second harmonic generation)

# Restructuring of IEL associated with elastin degredation

IEL

Collagen

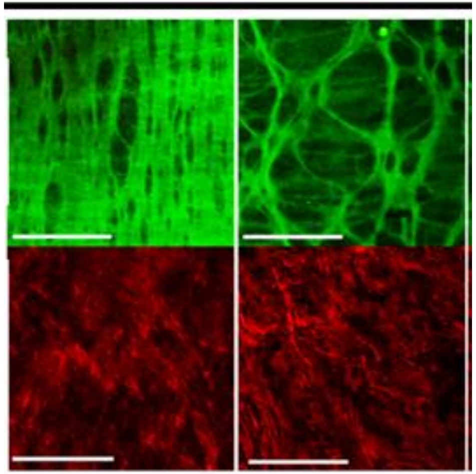

Sham FAL + AVF

Microfil casting of arterial tree

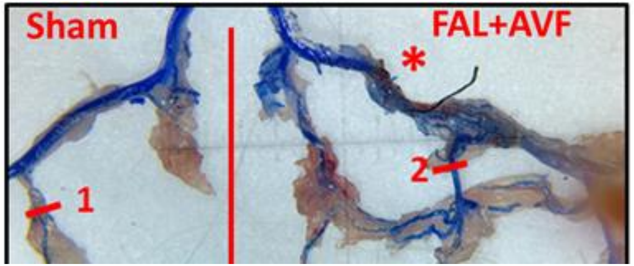


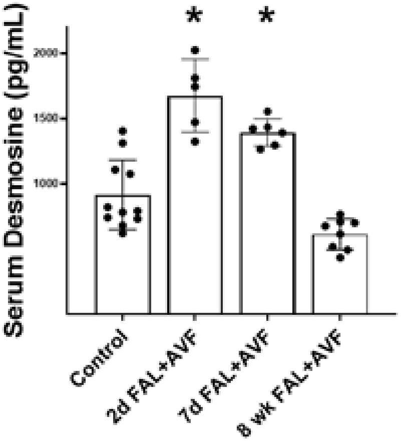


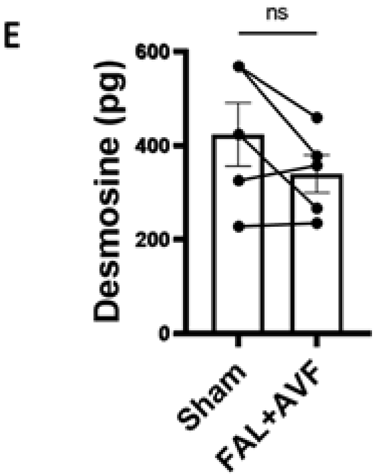

# Elastin crosslinking protein LOX is important in IEL integrity

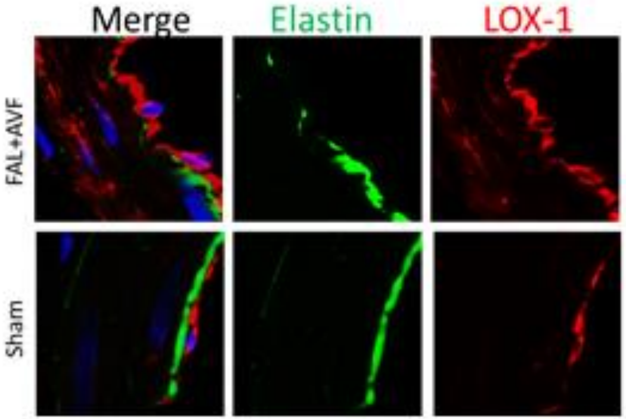


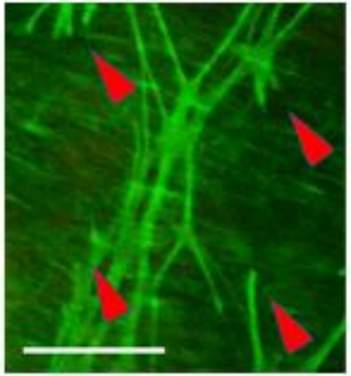

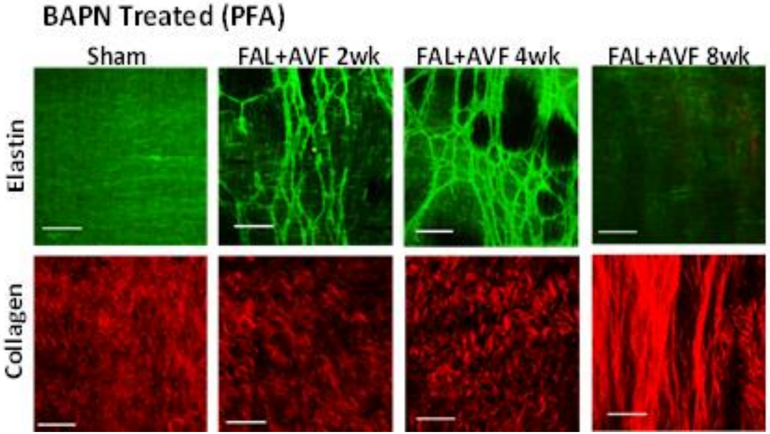

# Aim

- The aim of this study was to evaluate the balance of proteolysis with structural repair of the extracellular matrix during arterial remodeling

# Methods

8-12 week SD Rats → FAL + AVF → Tissues Collected at Interval Time Points → Microscopy, PCR + Serum Marker Analysis

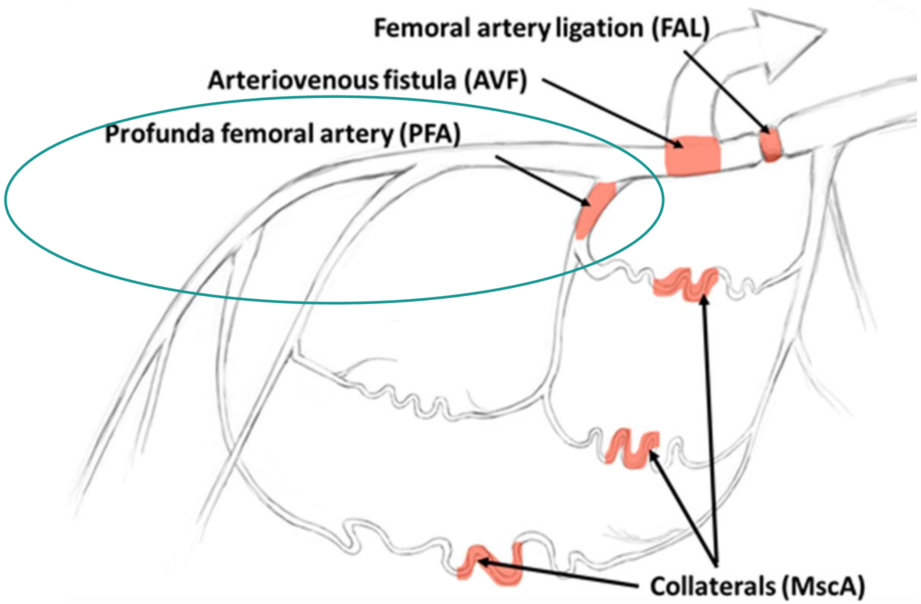

# Methods: Statistical Analysis

- Primers used for PCR included FBN-1, FBN-2, LOX-1, FBLN-4, FBLN-5, tropoelastin, MMP-2, and MMP-9, and were normalized to both GAPDH and the sham limb
  - mRNA fold changes were quantified using the $2^{-\Delta\Delta Cq}$ method

- Comparisons between time points were made with non-parametric ANOVA analysis with Bonferroni adjustment

# Results: Elastin degradation peaks early and returns to baseline after one week

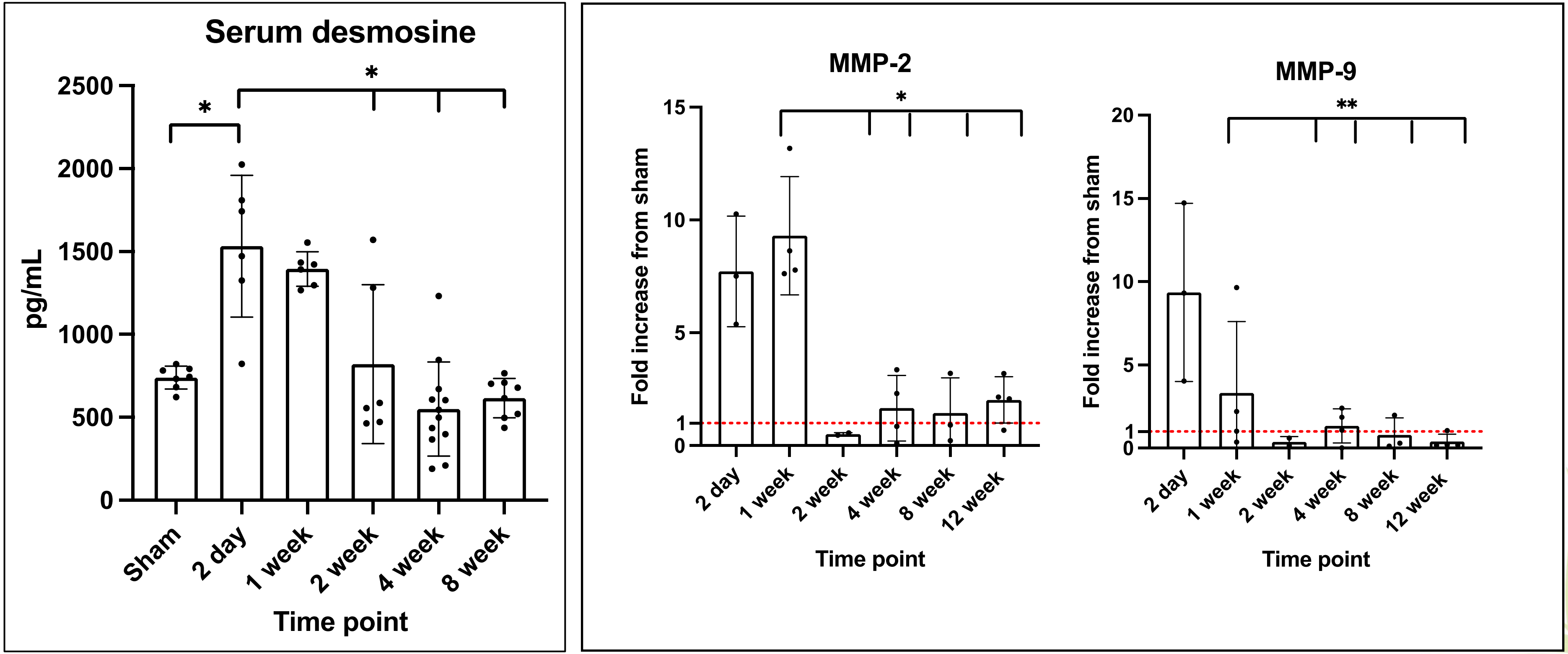

# Results: Early Upregulation in Extracellular Matrix Structural Proteins

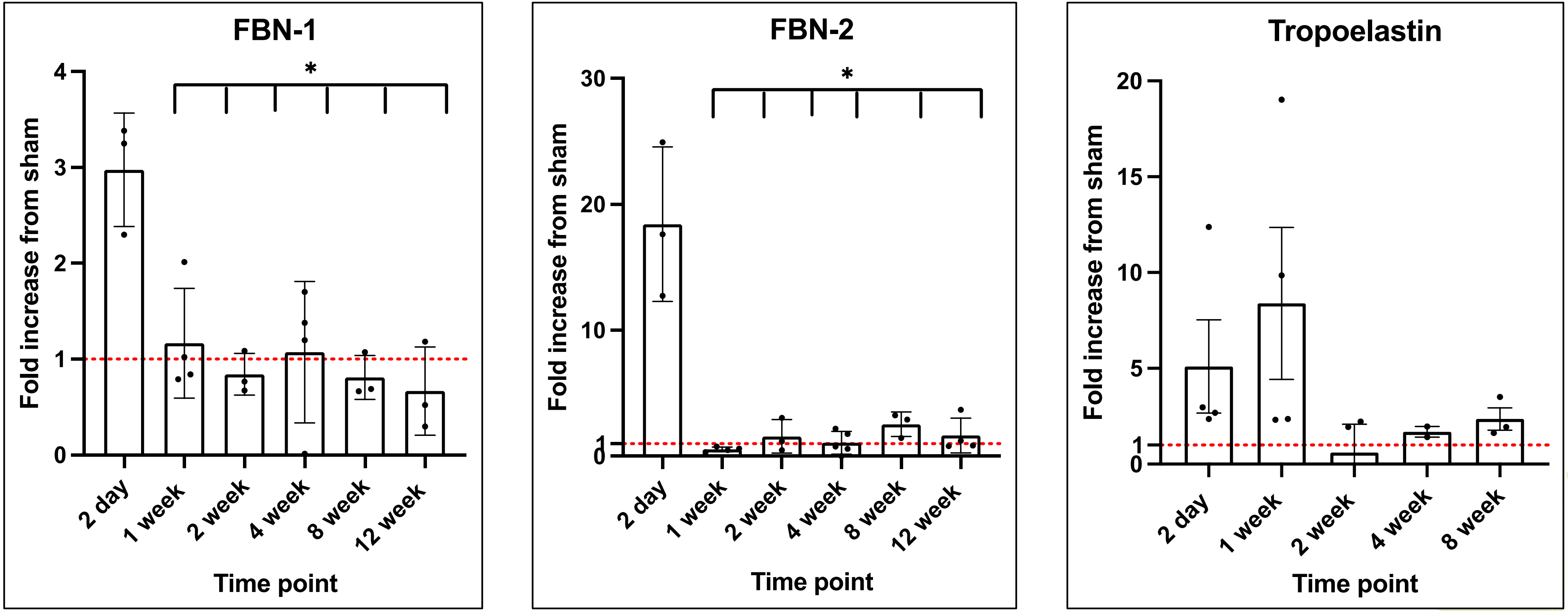

# Results: Early Upregulation in Extracellular Matrix Structural Proteins

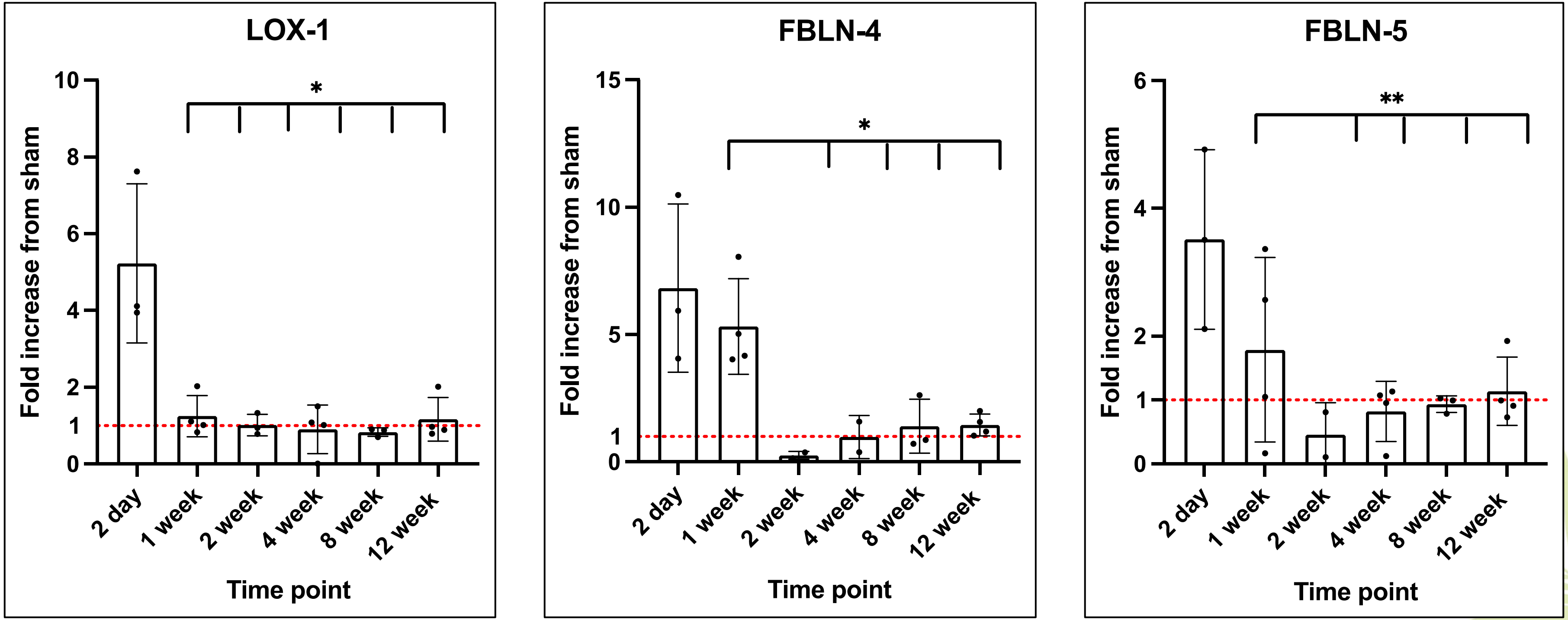

# Summary

- In arteriogenesis, elastic fibers within the extracellular matrix of arterial collaterals reorganize to support outward remodeling

- Elastic fiber degradation products initially increase but then return to baseline, suggesting an early peak in structural reorganization to allow for diameter increases

- These results suggest that outward remodeling must balance elastolysis to separate elastic fibers with simultaneous elastic fiber reinforcement to avoid irreversible fiber breakage and loss

# Thank you

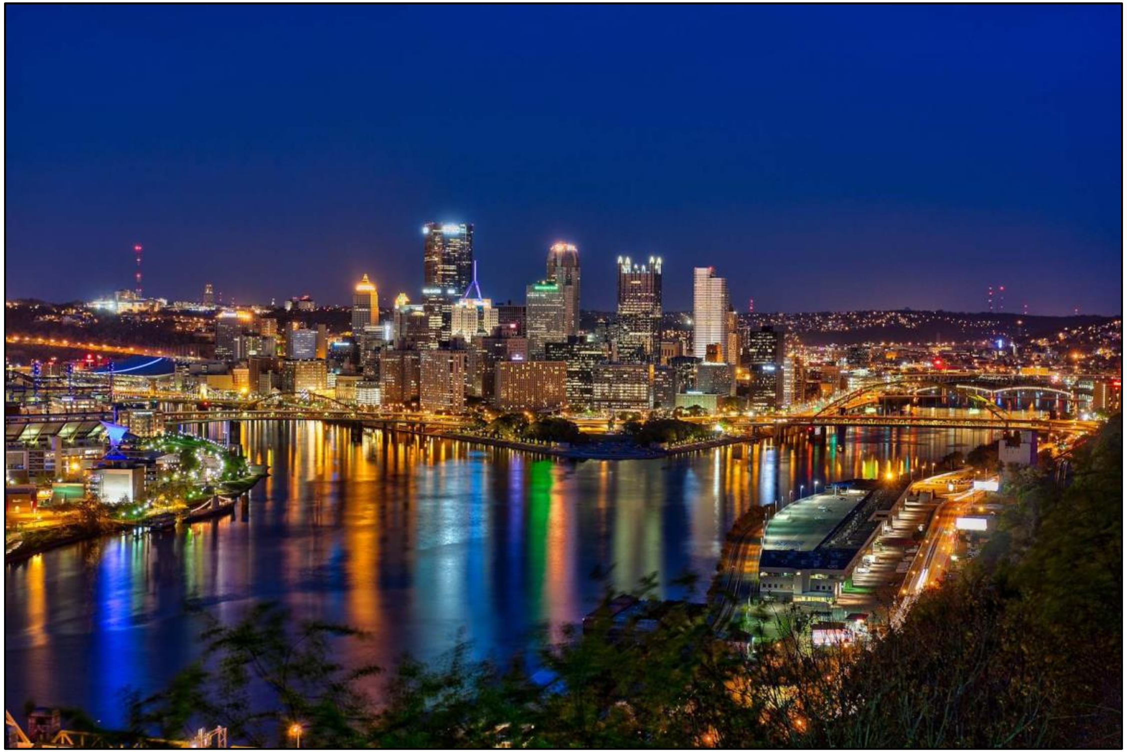

2021 VASCULAR ANNUAL MEETING

Rat PFA

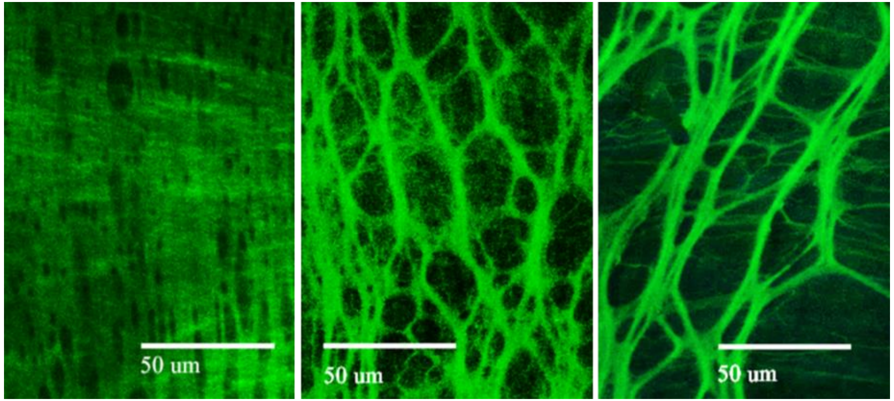


Human Collateral

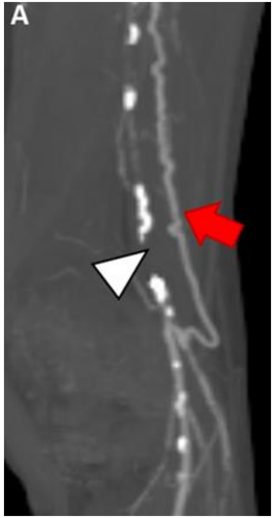


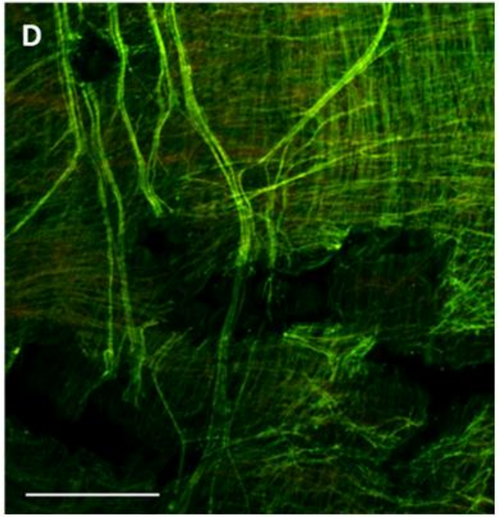

Supplement: Supplementary file 4 [file Presentation_1.pdf]
